# A comparison of hydrogen abstraction reaction between allyl-type monomers with thioxanthone-based photoinitiators without amine synergists

**DOI:** 10.3389/fchem.2022.967836

**Published:** 2022-09-02

**Authors:** Xiaotian Zhao, Wen Xu, Xi Chen, Shibo Lin, Xuanhao Li, Lihui He, Xu Liao, Guodong Ye

**Affiliations:** ^1^ Department of Pharmacy, Chengdu Second Peoples Hospital, Chengdu, China; ^2^ Department of Dermatology, Chengdu Second Peoples Hospital, Chengdu, China; ^3^ Guangzhou Municipal and Guangdong Provincial Key Laboratory of Molecular Target & Clinical Pharmacology, the NMPA and State Key Laboratory of Respiratory Disease, School of Pharmaceutical Sciences and the Fifth Affiliated Hospital, Guangzhou Medical University, Guangzhou, China

**Keywords:** allyl ether, allyl, thioxanthone, density functional theory, hydrogen abstraction, transition state

## Abstract

The photodriven radical-mediated [3 + 2] cyclization reaction was found to yield polymers efficiently without being hindered by degradative chain transfer. The first reaction is a hydrogen abstraction process in which one hydrogen atom migrates from the *α*-methylene group of an allyl monomer to the triplet state (or fragments) of the photoinitiator, thus yielding primary allyl radicals as primary radicals and then begins chain propagation *via* a 3 + 2 cyclization reaction. Allyl ether monomers were found to be significantly higher than other allyl monomers even with the absence of amine-like synergists. In order to clarify the procedure of the hydrogen abstraction mechanism, we used four allyl-type monomers as hydrogen donors and three thioxanthone photoinitiators as hydrogen acceptors by the quantum chemistry method in terms of geometry and energy. The results were interpreted with transition-state theory and the interaction/deformation model. Then, the tunneling factors of hydrogen abstraction reactions were also investigated by Eckart’s correction. The results show allyl ether systems are more reactive than other allyl systems, and it would provide us with new insights into these hydrogen abstractions.

## Introduction

The allyl-like compounds are characterized by the presence of the allyl group CH_2_ = CH-CH_2_-X-R (X = CH_2_, O, NH, OCO, S,… etc). Allyl monomers are also used in coating and copolymers based on them are used to improve the thermal stability and the resistance to wear of certain materials (Mark, 1978). Thiol-ene reactions are based on the CH_2_ = CH- with the help of initiators. Fenton reactions mainly focus on the adjacent CH_2_-applying cobalt driers (Yuan et al., 2004a; Yuan et al., 2004b). Polymers are also obtained by the insertion polymerization and copolymerization applications in engineering industries such as insulators, connecting sleeves, gas-tight seals, etc (Schildknecht, 1973). In our present work (Zhao et al., 2021a; Lun et al., 2022), we obtained the embolic microsphere based on allyl monomers, which has broad application prospects in embolization interventional therapy. In addition, the abovementioned wide range of applications of allyl polymers accounts for the interest in them in recent years (Herrero and Ullah, 2021).

In the past, allyl-like compounds polymerized with difficulty and gave polymers in low yields and with low molecular weights by thermopolymerization (Laible, 1958). However, in our early research, we used the photopolymerization of allyl ether monomers to obtain the embolic microsphere *via* a simple photodriven radical-mediated [3 + 2] cyclization reaction (PRMC) mechanism (Zhou et al., 2022). These mechanisms involve the cleavage of type I photoinitiators to yield radicals or the excited process of type II photoinitiators to yield triplet states in the first step. Subsequent abstraction of hydrogen from an allyl monomer promotes the formation of allyl radicals as primary radicals (Zhao et al., 2021b). Under irradiation, allyl radicals are excited and start a [3 + 2] cyclization reaction with the second allyl monomer and start chain-growth processes without being interrupted by degradative chain transfer (Curran, 2002). The degradative chain transfer is induced by the conjugated structure of three-center-three-electron in allyl radicals and causes a decrease in chain propagation. Compared to allyl ether monomer with other allyl-like monomers, it has extremely satisfied polymerization properties (Olivero et al., 2000).

Hydrogen abstraction (HAT) reactions are important initiation reactions generating primary allyl radicals. These HAT reactions could also be easily found in the free-radical polymerization of (meth)acrylates or autoxidation of alkyd resin using a drier (Dursun et al., 2003; Meereis et al., 2014), which also play a significant role in the first step. For example, the formation of the alkyl hydroperoxide is the “bottleneck” step in the autoxidation process, which reveals that polymerization requires a long time at the initial stage (Zhou et al., 2016). Now, this can be overcome by adding photoinitiators to increase the formation of the alkyl hydroperoxide. In addition, Yu et al. (2018) modified the combustion mechanism analysis of decalin developed by Dagaut et al. (2013), which indicated that hydrogen abstraction reactions are the important fuel consumption channels. Although hydrogen abstraction reactions are studied in many literature studies (Chen et al., 2021; Khiri et al., 2021), there are no available experimental and theoretical studies on the thermodynamic properties and kinetic parameters of hydrogen abstraction from allyl ether or other allyl monomers.

**FIGURE 1:**
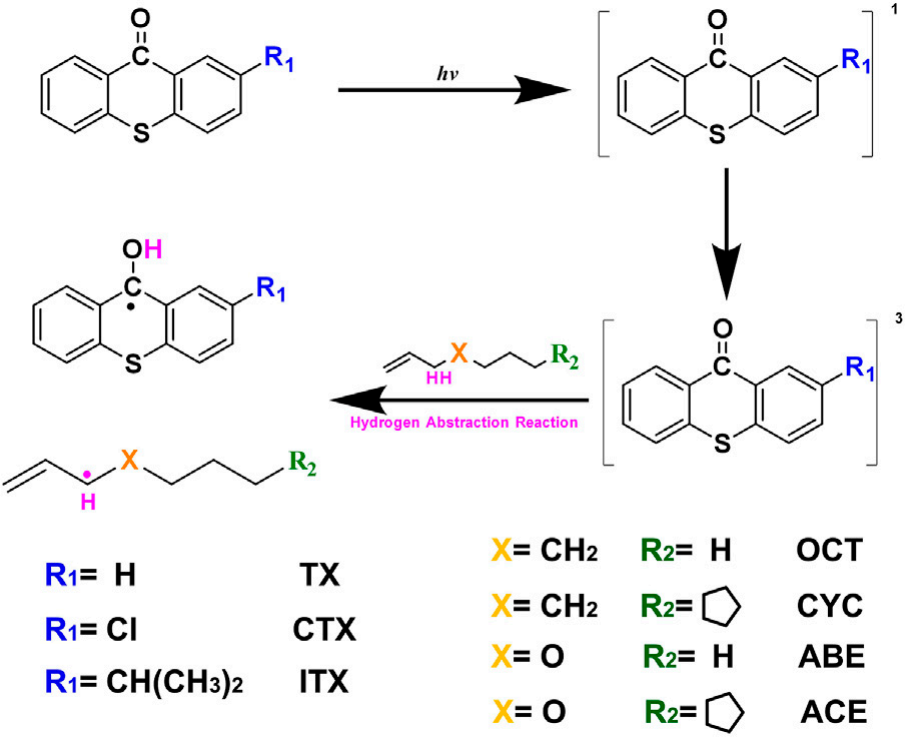
Mechanism of excited TXs as hydrogen acceptors reacting with C-H donors.

Aiming to provide accurate kinetic parameters and facilitate the modeling of allyl monomers, the high-precision quantum chemical method was used to study the hydrogen abstraction reactions. We used four allyl-like monomers as donors and TX series photoinitiators as acceptors. The molecular structure of our study is shown in Figure 1, and the four different monomers were selected to represent the allyl monomer, allyl ether monomer. Geometry, including the conformation of the donor and the geometry of the transition state (TS), and energy, including the transition state theory (TST) energy of donors and thermodynamic data (such as the activation energy, *E*_a_) of the reaction in combination with kinetic descriptors (such as rate constants, *k*), are obtained.

## Computational details

The donors were 1-octene (OCT), allyl butyl ether (ABE), 4-cyclopentane olefin (CYC), and allyl cyclopentane ether (ACE). The acceptors were three thioxanthone photoinitiators, including thioxanthone (TX), 2-chloro-thioxanthone (CTX), and 2-isopropylthioxanthone (ITX). The density functional theory (DFT) method (Perdew and Ruzsinszky, 2010) of theoretical calculations was used to identify and quantify the interactions between different molecules. Three thioxanthone initiators are selected as abstracting agents (TX, CTX, and ITX), and the corresponding groups of twelve reactions were labeled with TX + OCT, TX + ABE, and so on. Geometry optimization calculations of all species are performed employing B3LYP/6-311++g(d,p) level of theory. The reaction energies and barrier heights were evaluated using several density functional theory methods at the M06-2X/6-311++g(d,p) level (Zhao and Truhlar, 2008) and were scaled with a zero-point energy (ZPE) scale factor of 0.97 (Alecu et al., 2010) and using Gaussian 16 package (Gaussian, 2016) in the gas phase. It involved reactants, products, and transition state (TS). All the structures of stable molecules were confirmed to have no imaginary frequency. Meanwhile, there was only one imaginary frequency of the transition state (TS). The intrinsic reaction coordinate (IRC) (Schlegel, 2011) calculations were implemented to verify that the transition state connects the two right stationary points at 298.15 K and 1 atm. Gibb’s free energy (Δ_r_ *G*) and reaction enthalpies (Δ_r_ *H*) are obtained from the difference of reactants and products. The E_a_ is obtained from the difference of reactants and TS based on the data of free energy. The bond dissociation energy (BDE) (Blanksby and Ellison, 2003) and electrostatic potential (ESP) (Murray and Politzer, 2011) were obtained by Multiwfn 3.6 (Lu and Chen, 2012) and VMD 1.9 (Humphrey et al., 1996). The *k* and tunneling factors (*κ*(T)) were computed with KiSThelP (Canneaux et al., 2014). We also performed the *k* of these groups were independent of pressure. The high-pressure-limit *k* of twelve reactions is calculated by using the TST method with the consideration of *κ*(T). The detailed coordinates of compounds are shown in the Supplementary Material.

## Result and discussion

### Donors’ descriptors

**FIGURE 2:**
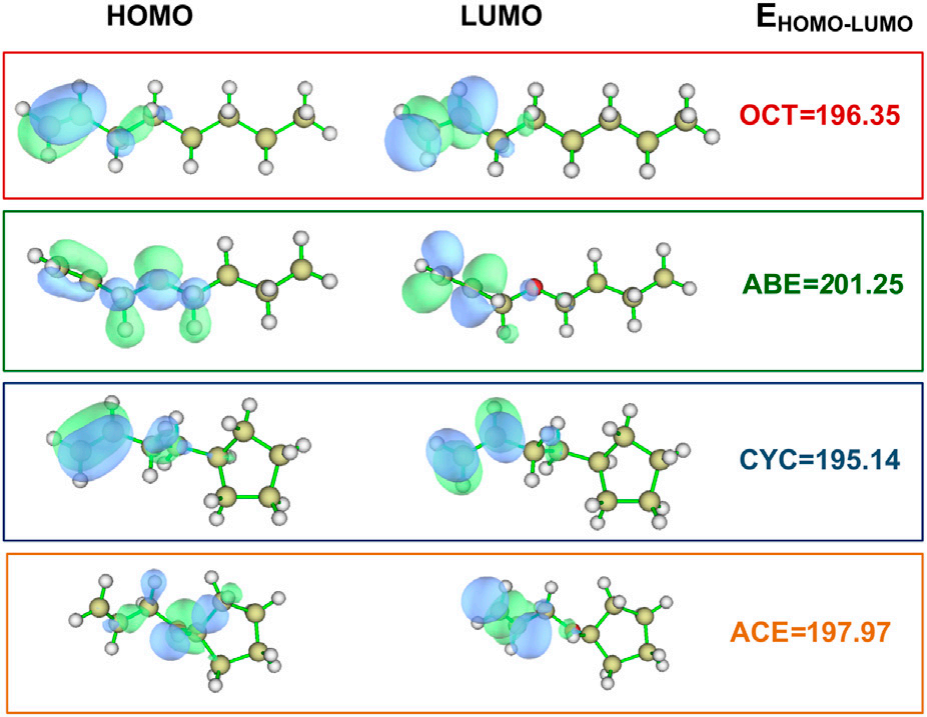
Values for HOMO and LUMO of the OCT, ABE, CYC, and ACE at the M06-2X/6-311++g(d,p) level (Unit: kcal/mol).

Two allyl ether monomers (ABE and ACE) combined with two allyl analogs (OCT and CYC) for comparison are performed with different electronic properties. It is a popular quantum mechanical descriptor for the highest occupied molecular orbital (HOMO) and the lowest unoccupied molecular orbital (LUMO) energies (Zhou and Parr, 1990). They play a significant role in governing a wide range of chemical interactions. The HOMO–LUMO energy gaps of the four monomers are evaluated and listed in Figure 2. The OCT compounds are alpha molecular orbital level (32) and alpha molecular orbital level (33), which depend on the HOMO–LUMO gap, respectively. The energy values of the HOMO (32) orbital and LUMO (33) orbital were laying at an energy value of -8.53 and -0.02 eV, respectively. The detailed data can be found in Supplementary Table S1. In comparing these HOMO–LUMO energy gaps of different compounds, the ABE shows the highest value of 201.25 kcal/mol. The higher HOMO–LUMO energy gap implies the kinetic energy is higher and has high chemical reactivity (*Saranya* et al., 2018). The result indicates that the allyl ether monomers are more highly reactive than the allyl analog.

**FIGURE 3:**
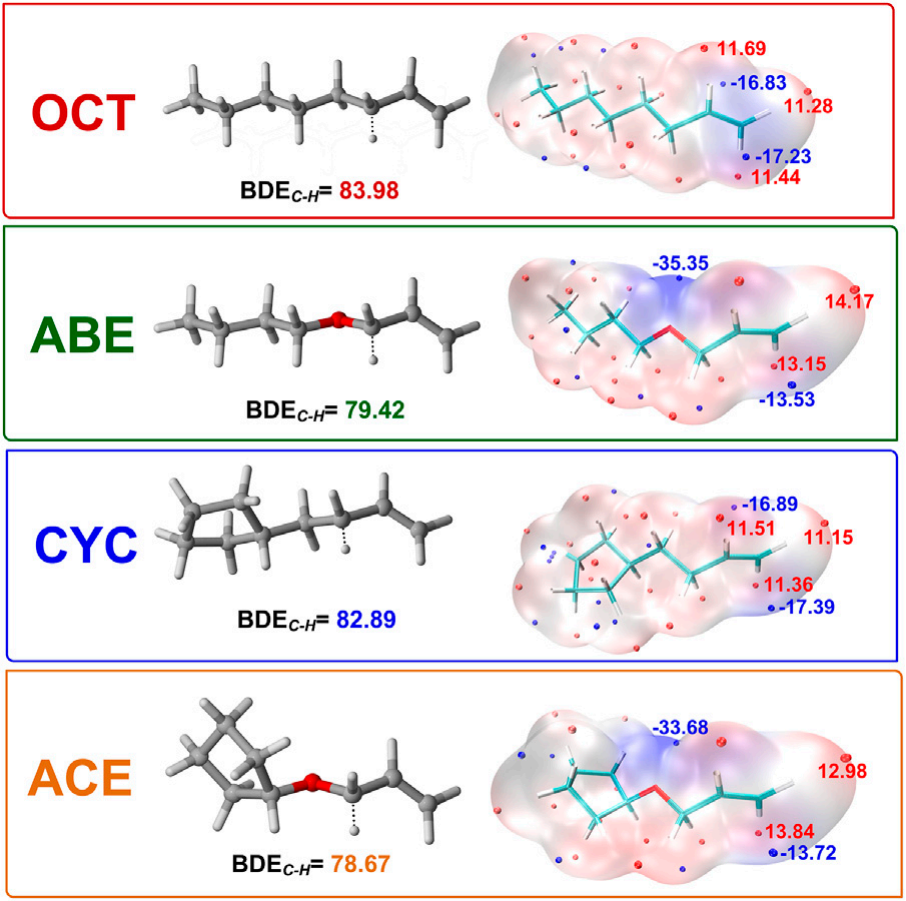
BDEs of C-H bonds in different donors and ESP-mapped molecular vdW surface of the four title monomers. Significant surface local minima and maxima of ESP are represented as red and blue spheres and labeled by red and blue texts, respectively (Unit: kcal/mol).

The BDE of C-H bonds in the donors is the key to hydrogen transfer (Zhou et al., 2016). As can be seen from the data in Figure 3, we found that ACE and ABE monomers have the lower BDE value at 78.67 and 79.42 kcal/mol, indicating two C-H bonds are more easily broken than others. The difference between the allyl and allyl ether was about 5 kcal/mol, implying that OCT and CYC do not actively participate in the HAT reaction.

The oxygen atom adjacent to double bonds has strong effects than the methylene group to decrease BDE. The lower BDE value provides clear evidence for our hypothesis that hydrogen abstractions have more easily proceeded as the first step at the beginning of PRMC.

The ESP picture is portrayed with the aim of studying the donor’s electronic character. It is critical for understanding and predicting intermolecular interaction to the ESP value (Politzer and Murray, 2002), which depicts the molecular surface electronic density. Moreover, its value is also helpful for us to explore the reactivity of four monomers as shown in Figure 3. The surface area is also displayed in different colors. The negative and positive ESP were represented by blue and red spheres, respectively. It can be seen in the allyloxy region of the ABE monomer that the surface minima of ESP are present between -O-CH_2_ –CH = CH_2_ carbon atoms as shown by the blue area, and the vdW surface has a large negative value of ESP around -35 kcal/mol. The allyl ether monomers possessing more negative ESP had a stronger ability to attract electrophiles and thus are more likely to be the reactive site. This is identical to the value of BDE.

### Transition state

**FIGURE 4:**
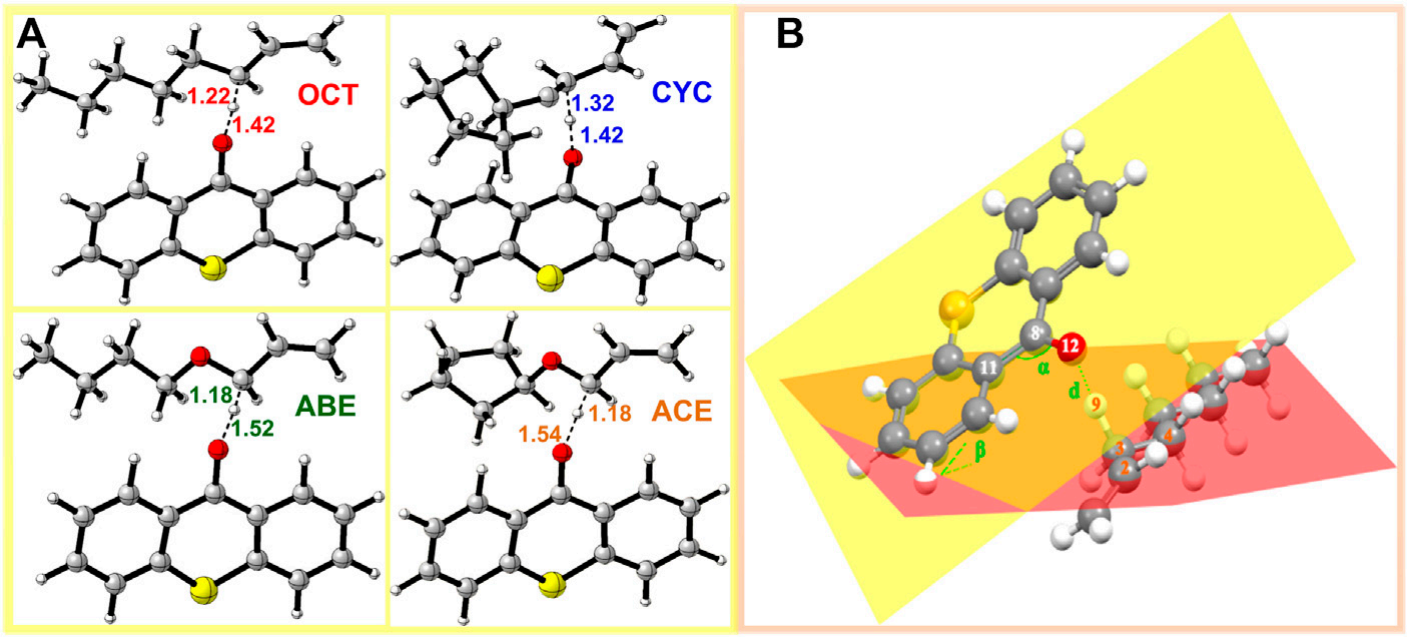
TS structure in HAT reactions: **(A)** structure of allyl monomers and allyl ether monomer complexes optimized at the level of B3LYP/6-311++G (d, p) (The dotted lines represent virtual σ bonds, and the unit is Å); **(B)** schematic view of the TS.

In order to investigate the TS of HAT using four monomers as reactants, we calculated twelve HAT reactions as shown in Figure 4. Three thioxanthone initiators are selected as abstracting agents (TX, CTX, and ITX), and the corresponding twelve reactions are labeled with TX + OCT, TX + ABE, and so on.

### Geometries

In the four systems, as shown in Figure 4, the central H_9_ atom migrates from the carbon atoms in the methyl chain (C_3_) to the oxygen atom (O_12_) in the triplet states of thioxanthone. The comparative results of the bond angles (Δα and Δβ) are displayed in Table 1, which also contains the corresponding distances of atoms. These distances for the O_12_-C_3_ in the TS are significantly longer than those in the ground states, which indicate that they appear ready to be broken for old C_3_-H_9_ bonds and formed for new O_12_-H_9_ bonds, respectively. In addition, there are performing the different d values in different groups (More details are shown in Supplementary Table S2). As is shown previously in Figure 4B, the Δα and Δβ are variations in the bond angle of ∠O_12_ = C_8_-C_11_ and the depression angle change of red and yellow planes (including the angles of the O_12_ = C_8_-C_11_ and C_2_-C_3_-C_4_ planes). According to these changes in deformation, it leads to an increase in the potential energy surface, which is generally defined as the deformation energy (E_d_). The interaction energy (E_i_) is from the difference between E_a_ and E_d_.

**TABLE 1:** Changes in bond angles and distances between the reaction complex (RC) and the transition state (TS).

Acceptor		Donor	Angle changes		Distance changes	
			Δα/°^a^	Δβ/°^b^	Δd/ $\overset{{}^{\circ}}{\text{A}}$ ^c^	Δd/%^d^
		OCT	−0.94	−0.49	0.45	31.69
		ABE	−1.33	−7.75	0.55	36.18
TX	+	CYC	−0.80	−1.85	0.44	31.21
		ACE	−1.10	−9.03	0.58	37.66
		OCT	−0.87	−0.49	0.46	32.17
CTX	+	ABE	−1.18	−7.84	0.57	37.25
		CYC	-0.83	−2.27	0.46	32.17
		ACE	−1.16	−8.51	0.59	37.82
		OCT	−0.87	2.87	0.42	30.22
ITX	+	ABE	−1.11	−7.46	0.51	34.46
		CYC	−0.86	−1.69	0.44	31.21
		ACE	−1.27	−8.64	0.57	37.25

a Δα = α_TS_−α_Prod_

b Δβ = β_TS_–β_Rct_

c Δd = d_TS_−d_Prod_

d Δd/% = Δd×100%d_TS_

As shown in Table 1, when allyl monomer reacted with triplet states of TX/CTX/ITX, almost no differences in Δα are present between the OCT and CYC, which values change below 0.1. It can be indicated that the deformations of the allyl monomers as reactants are nearly the same. However, the Δα value of ABE and ACE groups is greater, which shows that the deformation of allyl ether systems is larger than in allyl systems. These results were also validated by the Δβ values of four monomers, where the change in angle β with a value of 9.03^o^ for TX + ACE was the greatest one in Table 1. The Δβ for OCT and CYC groups is much lower than that of allyl ether systems.

According to Table 2, the E_d_ of the ITX + CYC group would be found to have the highest value (12.65) of the four monomers. However, the results of the angle change Δβ show that the TX + ACE group has a higher value (9.03) than that of the ITX + CYC group. In other words, the Δβ for planes we have chosen does not have a positive correlation as we expected in E_d_ section. Finally, it can be seen that the E_d_ of allyl ether groups is lower than that of allyl groups in Table 2.

**TABLE 2:** Thermodynamic parameters of twelve hydrogen abstraction reactions.

Acceptor		Donor	Δ_r_ *H*	Δ_r_ *G*	*E*_a_ = Kcal.mol^-1^	(*E*_d_+*E*_i_)			
						Sum=(Donor+Acceptor)			
			Kcal.mol^1^	Kcal.mol^−1^		Kcal.mol^−1^		Kcal.mol^−1^
		OCT	−22.31	−22.28	12.46	8.29	5.51	2.78	4.17
TX	+	ABE	−26.52	−26.62	10.47	5.30	2.82	2.48	5.44
		CYC	−22.67	−22.23	12.74	8.64	6.87	1.77	4.10
		ACE	−27.69	−27.81	11.09	6.06	3.18	2.88	5.04
		OCT	−21.18	−21.17	12.27	8.71	5.54	3.17	3.56
CTX	+	ABE	−25.40	−25.51	11.17	5.63	2.85	2.78	5.54
		CYC	−21.40	−21.55	14.02	11.06	6.48	4.58	2.97
		ACE	−26.42	−26.61	12.66	6.31	2.80	3.51	6.34
		OCT	−20.73	−20.74	14.06	9.63	6.48	3.15	4.43
ITX	+	ABE	−24.97	−25.13	11.86	6.96	3.92	3.04	4.90
		CYC	−21.26	−21.42	14.45	12.65	6.90	5.75	1.80
		ACE	−26.28	−26.49	12.84	9.11	3.21	5.90	3.73

Δ_r_ *H,* enthalpy change; Δ_r_ *G,* Gibbs’ free energy change/reaction drive force; *E*_a,_ activation energy; *E*_d,_ deformation energy; *E*_i,_ interaction energy.

We also obtained the bond length changes for the terminal atoms. Δd is the variation in bond length for O_12_-H_9_, and Δd% shows the magnitude of the change in the distance of the terminal oxygen. As is shown earlier, a comparison of the bond length reveals that ACE and ABE groups have a greater variation in bond length. In the CTX + ACE group, the bond length change reaches 37.82%, that is, 5% more than that in CYC groups. The values of allyl systems (OCT and CYC groups) are very close as above 31%. The greater Δd seems to indicate that the TS occurs earlier, and the earlier TS means that smaller activation energy (E_a_) is required for the reaction. The Δd% of OCT and CYC is very close, and they might, therefore, have similar energy barriers. According to the value E_a_ in Table 2, it was also validated by the allyl ether systems for the lower E_a_ value than the allyl systems.

### Energy

The thermodynamic properties of hydrogen abstraction reactions are obtained based on TST. According to the height difference in the potential energy surface, we can obtain E_a_. The E_d_ is the energy accompanying the structural distortion from the reaction complex to the TS in the IRC calculation (Ess and Houk, 2008). E_d_ is the difference value of E_a_ and E_d_ according to the distortion/interaction model as mentioned earlier. The Δ_r_H, Δ_r_G, E_a_, E_d,_ and E_i_ are summarized in Table 2.

The E_a_ values of allyl ether and allyl monomers are in the range of 10.47–12.84 and 12.27–14.06 kcal/mol, respectively. In the CYC groups, the E_a_ value of the ITX + CYC group is the largest, which reached 14.45 kcal/mol. However, the E_a_ for TX + ABE has taken the lowest value of 10.47 kcal/mol. For each group, the E_a_ height reactions are both in the order of CYC > OCT > ACE > ABE. Hence, one can expect that allyl ether monomers are more active among the HAT, and the corresponding reaction *k* is higher in Table 3.

**TABLE 3:** Imaginary frequencies, tunneling factors, rate constants, and bond orders of the twelve reactions.

Acceptor		Donor	ω ≠	*Κ*(T)	*k* cm^3^·molecule^−1^·s^−1^	*n*_T_
		OCT	−1358.30	2.21	1.82×10^−18^	0.26
		ABE	−919.96	1.12	1.78×10^−16^	0.22
TX	+	CYC	−1425.53	2.86	l.38×10^−19^	0.28
		ACE	−811.94	1.08	3.71×10^−17^	0.22
		OCT	−1307.51	2.05	1.40×10^−18^	0.27
CTX	+	ABE	−906.95	1.11	1.01×10^-−6^	0.23
		CYC	−1330.81	2.21	1.81×10^−19^	0.28
		ACE	−676.53	1.05	7.06×10^−17^	0.24
		OCT	−1581.69	3.78	1.00×10^−18^	0.29
ITX	+	ABE	−1248.53	1.37	8.32×10^−17^	0.24
		CYC	−1434.61	2.95	9.97×10^−20^	0.29
		ACE	−833.57	1.10	2.27×10^−17^	0.25

*κ*(T), tunneling coefficients; *k*, rate coefficients; *n*_T,_ bond order; ω ≠ , imaginary frequency.

Moreover, it reveals that the Δ_r_G (−26.61 kcal/mol) in the CTX + ACE group is much more negative than that in the CTX + CYC group (−21.55 kcal/mol), providing a huge reaction driving force. Comparison of the Δ_r_G values shows that allyl ether systems have a greater negative value than allyl systems, indicating that the allyl ether systems have more reactivity. The results of Δ_r_G and E_a_ are entirely consistent with the observations obtained by gel permeation chromatography and thermogravimetry-derivative thermogravimetry analysis earlier. We found the molecular weights and polymeric yields of allyl ether monomers were significantly higher than those of other ones (Chen et al., 2022).

The E_d_ values were affected not only by the bond angle changes but also by the bond length changes (Grabowski, 2011). As shown in Figure 4, the distance between the TX + ACE group and O_12_-H is 1.54 Å, which is the greatest distance of the four monomers. After this step, the distance of the product is shortened to 0.96. According to Table 1, the Δd reaches 0.58 Å, which is larger than the other groups, 0.45 Å for OCT, 0.55 Å for ABE, and 0.44 Å for CYC. It could be seen that the HAT in the allyl ether systems crosses the barrier from a greater distance. Generally, a smaller E_d_ value will result in a smaller E_a_, which favors the occurrence of the reaction.

### Rate constant

The *k* could help us to understand the kinetic nature of the reaction processes. It is calculated from the formula of conventional TST (Hu et al., 2010):

$$k=\sigma \frac{k_{\text{b}}T}{h}\cdot \frac{q^{\neq }}{q_{A}q_{B}}\cdot \exp\left(-\frac{E}{RT}\right),$$ (1)

where σ = 2 (for this reaction), which is the symmetry factor (degeneracy of the reaction path) that accounts for the two possible HAT from donors, *k*_b_ is the Boltzmann constant, T is the absolute temperature, *h* is Planck’s constant, *q* ^*≠*^, *q*_*A*_, and *q*_*B*_ are, respectively, the partition functions of the reactants A and B and the transition state per unit volume, and E is the classical height of the barrier. The kinetic parameters of twelve H-abstract reactions, for example, Eckart’s correction, are shown in Table 3. Eckart’s tunneling factor, *κ*(T), is expressed by the following Eq. 2:

$$\kappa \left(T\right)=1+\frac{1}{24}{\left(\frac{h\omega^{\neq }}{k_{\text{b}}T}\right)}^{2},$$ (2)

where *ω* ^≠^ is the imaginary frequency of the transition state, *T* is the temperature, *k*_b_ and *h* are the Boltzmann constant and Planck’s constant, respectively. Bond order, *n*_T_, is the criteria for ‘‘earliness” or ‘‘lateness” of the TS. The larger the value of *n*_T_, the later the TS would appear. According to the bond energy–bond order model (BEBO) (Blowers and Masel, 1998), *n*_T_ is obtained from Eq. 3:

$$n_{T}=\frac{E_{a}}{2E_{a}-\Delta H}.$$ (3)

According to the value of n_T_, we found the appearance of TS in TX + ABE is earlier than the TX + OCT. Peculiarly, the *ω* ^≠^ of the TX + ABE group is lower than that of TX + OCT, so the corresponding *κ*(T) becomes very low. It indicated that including allyl ether group reached the TS state earlier than the allyl group in HAT. According to our observation, the *κ*(T) value induced by radicals is usually over 3, and it has a pronounced impact on the reactivity (Liang et al., 2018). However, in our study, *κ*(T) values were near to 1 for the allyl ether system. For the allyl system, the *κ*(T) value became higher. We found *κ*(T) value increases with increasing E_a_. That means the low E_a_ is a significant factor in inducing this tunneling behavior. By comparing the *k* in twelve reactions, we found that the *k* value in the TX + ABE group reaction was 1.78 × 10^−16^ cm^3^∙molecule^−1^∙s^−1^ and is the highest. All the *k* values are reported in the form of a modified Arrhenius expression. The high-pressure limit *k* of these reactions for temperature varied from 500 to 2,500 K when treated with TST.

**FIGURE 5:**
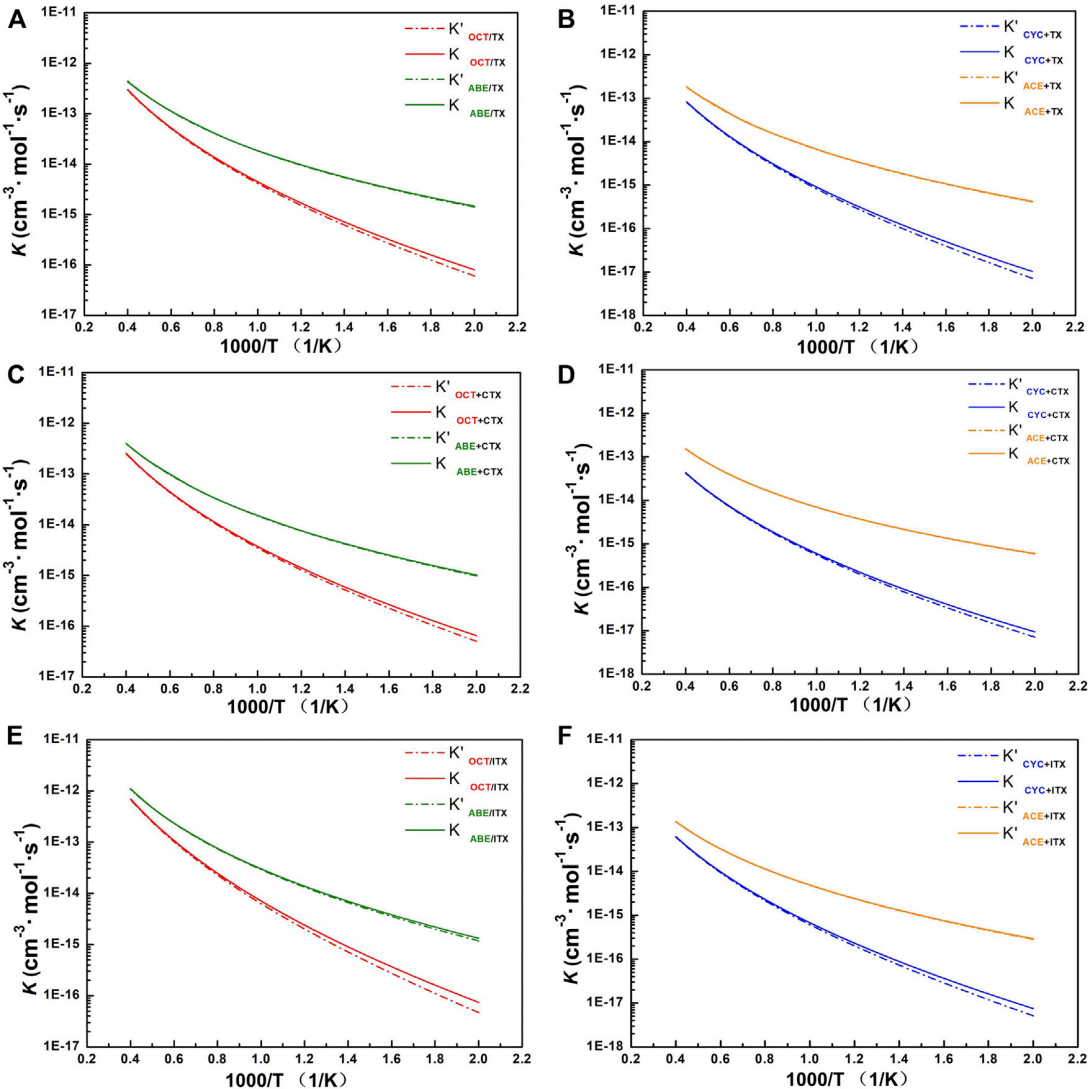
The profile of rate constant. **(A)** TX+OCT/ABE group. **(B)** TX+CYC/ACE group. **(C)** CTX+OCT/ABE group. **(D)** CTX+CYC/ACE group. **(E)** ITX+OCT/ABE group. **(F)** ITX+CYC/ACE group. *k* with and without tunneling correction as a function of temperature from 500 to 2,500 K. Dash line: rate constant without tunneling correction. Solid line: rate constant obtained with Eckart’s method.

It can be seen from Figure 5 that the computational *k* for involved allyl ether (ABE/ACE) reactions are higher than those for allyl (OCT/CYC) reactions at the same temperature. It also shows a weaker negative temperature dependence for allyl ether reactions than the others. At 500 K, the *k* of ABE reactions is higher than that of ACE reactions since the former owns the lowest energy barriers in each group, but until 2,500 K, it can be seen that *k* in ACE reactions is similar to that in ABE. When ABE/ACE react with the same acceptor, the two reactions tend to possess similar *k* as a result of the small difference among their barrier heights. In addition, these reactions' *k* values with Eckart’s method (solid line) are relatively close to the without tunneling correction (dash line).

For group TX + OCT/ABE, the change of our computed value for the OCT and ABE reactions’ constant rates is one to three orders of magnitude of the deviations in the temperature range of 500–1,000 K. Especially, the rate line of OCT reactions is sharper than that of ABE, and it indicates that reaction rate change of OCT is more sensitive at the beginning temperature-up. At the 500 K, the ABE reaction rate is 1.46 × 10^−15^, and the OCT reaction rate is 8.02 × 10^−17^. Until the 2,500 K, these values are similar to 4.35 × 10^−13^ and 3.02 × 10^−13^, respectively.

For group TX + CYC/ACE, the *k* of the TX + ACE reaction shows two orders of magnitude of the TX + CYC reaction at 500 K. While at the 2,500 K, the value of *k* for the TX + ACE reaction is one order of magnitude of the TX + CYC reaction. Comparing the reactions of the other acceptors (CTX and ITX), the tendency of values is similar to that of TX reactions, owing to these thioxanthone-based structures. More details can be found in Supplementary Table S3.

For the ITX + ABE reaction, our computational *k* of it is almost the highest over the entire temperature range, followed by reactions TX + ABE and CTX + ABE. Especially at 1,500 K, its value is one order of magnitude of the other. Moreover, by comparing the ABE structure to other donors, its computational rate constant value is found to be the highest between 500 and 2,500 K.

According to Supplementary Table S3, it revealed that for allyl ether monomers as donors, the *k* value is higher than that of allyl monomers, indicating that the allyl ether structure is more reaction effective. In addition, the tendency of all reaction rate values is in conformity with temperature.

## Conclusion

Based on the result of the calculation, two types of monomers, allyl vs*.* allyl ether, have different chemical properties. By comparing the reactions of TSs with allyl or allyl ether, we found that the allyl ether system has more chemical reactivity in HAT. The allyl ether monomer processed not only higher electron density but also lower BDE than allyl systems. HAT in allyl ether monomer is more easily reacted according to lower E_a_. Moreover, according to the *k*, the allyl ether system has a higher value and has a positive correlation with temperature. In our research earlier, we proposed the PRMC mechanism in the polymerization of allyl ether, and the hydrogen abstraction is the first step in the polymeric process. Meanwhile, our calculation works are of paramount values and fairly contribute to the building of the polymerization mechanism of allyl ether in the future.

## Data Availability

The original contributions presented in the study are included in the article/Supplementary Material: further inquiries can be directed to the corresponding author.
