# Supplementary material for: A comparison of hydrogen abstraction reaction between allyl-type monomers with thioxanthone-based photoinitiators without amine synergists
Source: Front Chem. 2022 Sep 2;10:967836. doi: 10.3389/fchem.2022.967836 (PMC9478512; doi:10.3389/fchem.2022.967836)
Supplement: Supplementary file 1 [file DataSheet1.docx]

# *Supplementary Material*

# A comparison of hydrogen abstraction reaction between allyl-type monomers with thioxanthone based photoinitiators without amine synergists

Xiaotian Zhao ^1†^, Wen Xu ^2†^, Xi Chen ^1^, Shibo Lin ^1^, Xuanhao Li ^1^, Lihui He ^1^, Xu Liao ^1^, Guodong Ye* ^3^

^1^ *Department of Pharmacy, Chengdu Second Peoples Hospital, Chengdu 610017, P.R. China*

*^2^ Department of Dermatology, Chengdu Second Peoples Hospital, Chengdu 610017, P.R. China*

^3^ *Guangzhou Municipal and Guangdong Provincial Key Laboratory of Molecular Target & Clinical Pharmacology, the NMPA and State Key Laboratory of Respiratory Disease, School of Pharmaceutical Sciences and the Fifth Affiliated Hospital, Guangzhou Medical University, Guangzhou 511436, China*

† *These authors have contributed equally to this work*

***Correspondence**:

Guodong Ye
Tel: +86-13229494539
email: [gzhygd@gzhmu.edu.cn](mailto:gzhygd@gzhmu.edu.cn)

**Cartesian coordinates the optimized structures**

OCT

| molecular  H |  | x  -0.22126 | y  1.998569 | z  0.867489 |
| --- | --- | --- | --- | --- |
| C |  | -0.38339 | 4.01395 | 1.123627 |
| C |  | 0.584102 | 4.900538 | 0.832647 |
| H |  | -0.97198 | 4.161994 | 2.027705 |
| H |  | 1.202344 | 4.793406 | -0.05313 |
| H |  | 0.775661 | 5.758773 | 1.466275 |
| C |  | -0.70092 | 2.822944 | 0.327132 |
| H |  | -0.19262 | 2.819386 | -0.64215 |
| C |  | -2.4417 | 1.19827 | -0.62617 |
| H |  | -1.89534 | 0.346912 | -0.20698 |
| H |  | -2.03536 | 1.359947 | -1.63284 |
| C |  | -3.92939 | 0.845897 | -0.73281 |
| H |  | -4.47938 | 1.7023 | -1.14527 |
| H |  | -4.33539 | 0.680468 | 0.273742 |
| C |  | -4.2033 | -0.39165 | -1.59508 |
| H |  | -3.65413 | -1.24596 | -1.18224 |
| H |  | -3.79757 | -0.22665 | -2.60071 |
| C |  | -5.69093 | -0.74069 | -1.69686 |
| H |  | -6.26203 | 0.082475 | -2.13821 |
| H |  | -5.85194 | -1.62628 | -2.31796 |
| H |  | -6.11751 | -0.94604 | -0.70982 |
| C |  | -2.17637 | 2.442124 | 0.227765 |
| H |  | -2.58135 | 2.294954 | 1.236413 |
| H |  | -2.72414 | 3.296029 | -0.1971 |
| ABE  molecular |  | X | Y | Z |
| H |  | -1.90277914 | 1.217671 | 1.035461 |
| C |  | -3.24658214 | -0.27847 | 0.342524 |
| C |  | -4.25332214 | -0.29791 | -0.52244 |
| H |  | -3.31407514 | -0.89289 | 1.238217 |
| H |  | -4.23320314 | 0.295251 | -1.43043 |
| H |  | -5.13531914 | -0.90341 | -0.35574 |
| C |  | -1.98633914 | 0.518793 | 0.19385 |
| H |  | -2.04198114 | 1.127952 | -0.71332 |
| C |  | 0.34571486 | 0.527943 | 0.067035 |
| H |  | 0.40567086 | 1.216893 | 0.917559 |
| H |  | 0.30758686 | 1.157393 | -0.82951 |
| C |  | 1.60865286 | -0.33244 | 0.024777 |
| H |  | 1.54870986 | -1.0216 | -0.82559 |
| H |  | 1.64775186 | -0.96232 | 0.921202 |
| C |  | 2.90122086 | 0.478237 | -0.076 |
| H |  | 2.96171186 | 1.166313 | 0.774 |
| H |  | 2.86246486 | 1.107242 | -0.97179 |
| C |  | 4.15768086 | -0.38945 | -0.1181 |
| H |  | 4.14306286 | -1.06405 | -0.9779 |
| H |  | 5.06123786 | 0.219306 | -0.19001 |
| H |  | 4.24308186 | -1.00467 | 0.781226 |
| O |  | -0.82811958 | -0.21131 | 0.158012 |
|  |  |  |  |  |

CYC

molecular

| C |  | x  3.349363 | y  -0.13735 | z  -0.38756 |
| --- | --- | --- | --- | --- |
| C |  | 4.461218 | 0.242379 | 0.226932 |
| H |  | 3.340546 | -0.27667 | -1.4645 |
| H |  | 4.492805 | 0.400801 | 1.299279 |
| H |  | 5.382695 | 0.406511 | -0.317 |
| C |  | 2.054549 | -0.42288 | 0.302935 |
| H |  | 1.8441 | -1.50061 | 0.247716 |
| H |  | 2.118749 | -0.15374 | 1.366301 |
| C |  | 0.465714 | 0.71716 | -0.68035 |
| C |  | -0.46863 | -0.21502 | -1.43827 |
| C |  | -0.36118 | 1.476729 | 0.347221 |
| H |  | 0.960988 | 1.430852 | -1.38585 |
| C |  | -1.88724 | 0.0497 | -0.95502 |
| H |  | -0.18745 | -1.28236 | -1.25289 |
| H |  | -0.38935 | -0.04076 | -2.54045 |
| C |  | -1.81337 | 1.048088 | 0.191195 |
| H |  | 0.00186 | 1.255837 | 1.382403 |
| H |  | -0.2597 | 2.580164 | 0.191786 |
| H |  | -2.5117 | 0.460154 | -1.78828 |
| H |  | -2.37177 | -0.9007 | -0.61825 |
| H |  | -2.46376 | 1.934108 | -0.01822 |
| H |  | -2.18656 | 0.583086 | 1.13824 |
| C |  | 1.27785 | 0.134426 | -0.17774 |
| H |  | 1.838263 | 0.462768 | 0.672571 |
| H |  | 1.614292 | -0.83744 | -0.47302 |

ACE

Molecular

| C |  | x  2.699701 | y  -0.30735 | z  -0.25124 |
| --- | --- | --- | --- | --- |
| C |  | 3.344397 | 0.735593 | -0.75511 |
| H |  | 2.837504 | -1.29265 | -0.69121 |
| H |  | 3.221125 | 1.730954 | -0.34078 |
| H |  | 4.023145 | 0.637836 | -1.59252 |
| C |  | 1.773513 | -0.25759 | 0.935842 |
| H |  | 2.207479 | -0.81654 | 1.774564 |
| H |  | 1.648014 | 0.782835 | 1.264994 |
| C |  | -0.34553 | -0.23044 | -0.2357 |
| C |  | -1.56043 | -1.15604 | -0.45108 |
| C |  | -0.89695 | 1.139064 | 0.240847 |
| H |  | 0.219135 | -0.09785 | -1.16732 |
| C |  | -2.83785 | -0.29564 | -0.3984 |
| H |  | -1.55616 | -1.90227 | 0.346678 |
| H |  | -1.47959 | -1.69388 | -1.3969 |
| C |  | -2.43982 | 1.081961 | 0.187214 |
| H |  | -0.55459 | 1.338308 | 1.258707 |
| H |  | -0.51302 | 1.949252 | -0.38186 |
| H |  | -3.27624 | -0.1847 | -1.39205 |
| H |  | -3.59887 | -0.77402 | 0.220483 |
| H |  | -2.83739 | 1.894427 | -0.42332 |
| H |  | -2.86337 | 1.214245 | 1.184466 |
| O |  | 0.512583 | -0.88042 | 0.712728 |

01.TX+OCT group TS in Hydrogen Abstraction Reaction

| H | x  -0.19459 | y  1.952741 | z  0.897528 |
| --- | --- | --- | --- |
| C | -1.26155 | -2.92666 | 2.085877 |
| C | -0.77735 | -4.00954 | 1.338171 |
| C | 0.241585 | -3.80071 | 0.414558 |
| C | 0.781488 | -2.52659 | 0.233758 |
| C | 0.314571 | -1.41136 | 0.990177 |
| C | -0.72945 | -1.66166 | 1.920478 |
| C | 0.865513 | -0.09448 | 0.858576 |
| S | 1.97842 | -2.35002 | -1.04857 |
| C | 2.64223 | -0.73424 | -0.79929 |
| C | 2.031719 | 0.206022 | 0.08052 |
| O | 0.334442 | 0.86312 | 1.635934 |
| C | 2.650399 | 1.480491 | 0.175942 |
| C | 3.777204 | -0.40453 | -1.54113 |
| C | 4.344612 | 0.86121 | -1.43706 |
| H | -2.05928 | -3.08158 | 2.803855 |
| H | -1.18977 | -5.00208 | 1.473484 |
| H | 0.623164 | -4.63002 | -0.17119 |
| H | -1.09121 | -0.82662 | 2.50454 |
| H | 2.230023 | 2.196837 | 0.868612 |
| H | 4.217639 | -1.14555 | -2.19958 |
| H | 5.225853 | 1.109487 | -2.01597 |
| C | -0.41005 | 4.059778 | 1.093588 |
| C | 0.557437 | 4.946366 | 0.802608 |
| H | -0.99864 | 4.207822 | 1.997666 |
| H | 1.175679 | 4.839234 | -0.08317 |
| H | 0.748996 | 5.804601 | 1.436236 |
| C | -0.72758 | 2.868772 | 0.297093 |
| H | -0.21929 | 2.865214 | -0.67219 |
| C | -2.46836 | 1.244098 | -0.65621 |
| H | -1.92201 | 0.39274 | -0.23701 |
| H | -2.06202 | 1.405775 | -1.66287 |
| C | 3.774611 | 1.798173 | -0.5643 |
| H | 4.226293 | 2.778169 | -0.45611 |
| C | -3.95605 | 0.891725 | -0.76285 |
| H | -4.50605 | 1.748128 | -1.17531 |
| H | -4.36206 | 0.726296 | 0.243703 |
| C | -4.22997 | -0.34582 | -1.62512 |
| H | -3.6808 | -1.20013 | -1.21227 |
| H | -3.82424 | -0.18082 | -2.63075 |
| C | -5.7176 | -0.69486 | -1.7269 |
| H | -6.28869 | 0.128303 | -2.16825 |
| H | -5.8786 | -1.58045 | -2.348 |
| H | -6.14417 | -0.90022 | -0.73986 |
| C | -2.20303 | 2.487952 | 0.197726 |
| H | -2.60801 | 2.340782 | 1.206374 |
| H | -2.75081 | 3.341857 | -0.22714 |

02.TX+ABE group TS in Hydrogen Abstraction Reaction

| H | x  -0.53932 | y  1.83597 | z  0.869313 |
| --- | --- | --- | --- |
| C | -0.82483 | -3.28148 | 1.790667 |
| C | -0.13968 | -4.22339 | 1.009728 |
| C | 0.877343 | -3.79672 | 0.161958 |
| C | 1.216377 | -2.44461 | 0.090124 |
| C | 0.543064 | -1.46942 | 0.882715 |
| C | -0.49138 | -1.94063 | 1.733578 |
| C | 0.881206 | -0.07416 | 0.863161 |
| S | 2.431213 | -1.99759 | -1.10696 |
| C | 2.814533 | -0.31735 | -0.72925 |
| C | 2.019827 | 0.454869 | 0.168042 |
| O | 0.164749 | 0.735479 | 1.647102 |
| C | 2.419621 | 1.802695 | 0.370045 |
| C | 3.915783 | 0.234569 | -1.38466 |
| C | 4.266736 | 1.564504 | -1.176 |
| H | -1.6219 | -3.6065 | 2.45026 |
| H | -0.39606 | -5.27464 | 1.061273 |
| H | 1.414375 | -4.51497 | -0.44814 |
| H | -1.00679 | -1.21291 | 2.345781 |
| H | 1.856502 | 2.395734 | 1.077984 |
| H | 4.500318 | -0.38307 | -2.05809 |
| H | 5.123174 | 1.986027 | -1.68801 |
| C | -1.10708 | 3.909072 | 0.94224 |
| C | -0.23031 | 4.875943 | 0.658097 |
| H | -1.81144 | 4.025942 | 1.761334 |
| H | 0.483225 | 4.778972 | -0.15414 |
| H | -0.20063 | 5.795736 | 1.23017 |
| C | -1.17098 | 2.611485 | 0.234623 |
| H | -0.67338 | 2.619341 | -0.74633 |
| O | -2.48076 | 2.142859 | 0.161537 |
| C | -2.6458 | 0.937847 | -0.59309 |
| H | -2.04891 | 0.142802 | -0.13062 |
| H | -2.26616 | 1.097793 | -1.61241 |
| C | 3.512055 | 2.340832 | -0.28533 |
| H | 3.795146 | 3.370363 | -0.09524 |
| C | -4.12046 | 0.571728 | -0.61336 |
| H | -4.68725 | 1.405318 | -1.04253 |
| H | -4.46627 | 0.452614 | 0.41912 |
| C | -4.39317 | -0.71139 | -1.40747 |
| H | -3.81103 | -1.5355 | -0.97901 |
| H | -4.03498 | -0.58689 | -2.43655 |
| C | -5.87623 | -1.09234 | -1.4292 |
| H | -6.48138 | -0.30203 | -1.88408 |
| H | -6.04209 | -2.00917 | -2.00116 |
| H | -6.25631 | -1.25855 | -0.41653 |

03.CTX+OCT group TS in Hydrogen Abstraction Reaction

| H | x  0.195148 | y  1.735597 | z  0.987914 |
| --- | --- | --- | --- |
| C | -2.919316 | -2.22516 | 2.06729 |
| C | -2.944401 | -3.39566 | 1.2965 |
| C | -1.924365 | -3.63435 | 0.382035 |
| C | -0.880626 | -2.71919 | 0.234437 |
| C | -0.82637 | -1.52847 | 1.014346 |
| C | -1.886217 | -1.31586 | 1.933618 |
| C | 0.245942 | -0.57704 | 0.917625 |
| S | 0.292444 | -3.06134 | -1.0369 |
| C | 1.580411 | -1.89146 | -0.75958 |
| C | 1.42989 | -0.79864 | 0.144586 |
| O | 0.173216 | 0.500809 | 1.707618 |
| C | 2.533083 | 0.086478 | 0.271498 |
| C | 2.74983 | -2.05872 | -1.50058 |
| C | 3.807431 | -1.16304 | -1.38153 |
| H | -3.714672 | -2.03076 | 2.77814 |
| H | -3.750033 | -4.11149 | 1.406583 |
| H | -1.934218 | -4.53559 | -0.22131 |
| H | -1.855384 | -0.41752 | 2.534787 |
| H | 2.468971 | 0.896252 | 0.983432 |
| H | 2.837774 | -2.89924 | -2.18023 |
| H | 4.713399 | -1.28902 | -1.95819 |
| C | 0.908993 | 3.71769 | 1.255 |
| C | 2.18682 | 4.079022 | 1.039568 |
| H | 0.398976 | 4.103525 | 2.136293 |
| H | 2.745275 | 3.71311 | 0.183943 |
| H | 2.704545 | 4.753157 | 1.71203 |
| C | 0.140242 | 2.802217 | 0.405491 |
| H | 0.639263 | 2.595707 | -0.54655 |
| C | -2.090138 | 2.113213 | -0.6576 |
| H | -1.989491 | 1.102977 | -0.24693 |
| H | -1.608731 | 2.094359 | -1.64377 |
| C | 3.674444 | -0.10196 | -0.47902 |
| C | -3.576142 | 2.447383 | -0.82745 |
| H | -3.6783 | 3.462553 | -1.2335 |
| H | -4.058595 | 2.464793 | 0.158594 |
| C | -4.323057 | 1.463831 | -1.73559 |
| H | -4.220663 | 0.450727 | -1.32913 |
| H | -3.840953 | 1.446341 | -2.72068 |
| C | -5.807689 | 1.801038 | -1.90084 |
| H | -5.94194 | 2.795663 | -2.33804 |
| H | -6.310548 | 1.08161 | -2.5531 |
| H | -6.325171 | 1.792283 | -0.93622 |
| C | -1.348092 | 3.104208 | 0.24457 |
| H | -1.821074 | 3.134143 | 1.23366 |
| H | -1.449838 | 4.117418 | -0.17086 |
| Cl | 5.020435 | 1.016776 | -0.28787 |

04.CTX+ABE group TS in Hydrogen Abstraction Reaction

| H | X  0.053415 | Y  -1.73262 | Z  0.906928 |
| --- | --- | --- | --- |
| C | 2.721943 | 2.66178 | 1.839274 |
| C | 2.573452 | 3.812432 | 1.052601 |
| C | 1.486601 | 3.915596 | 0.191294 |
| C | 0.548916 | 2.883986 | 0.112546 |
| C | 0.671571 | 1.710743 | 0.909804 |
| C | 1.79367 | 1.638188 | 1.77395 |
| C | -0.28686 | 0.636938 | 0.884494 |
| S | -0.72133 | 3.066716 | -1.09675 |
| C | -1.84602 | 1.757639 | -0.74456 |
| C | -1.52353 | 0.705804 | 0.164829 |
| O | -0.04973 | -0.409 | 1.672004 |
| C | -2.50618 | -0.30234 | 0.353979 |
| C | -3.06084 | 1.773641 | -1.42884 |
| C | -3.99773 | 0.761271 | -1.24804 |
| H | 3.569717 | 2.57302 | 2.509488 |
| H | 3.296161 | 4.617341 | 1.110121 |
| H | 1.360978 | 4.800449 | -0.42324 |
| H | 1.895009 | 0.754546 | 2.389515 |
| H | -2.31128 | -1.08614 | 1.071517 |
| H | -3.28 | 2.58622 | -2.11284 |
| H | -4.93882 | 0.769589 | -1.78037 |
| C | -0.40757 | -3.82526 | 1.049292 |
| C | -1.65075 | -4.26111 | 0.824459 |
| H | 0.188786 | -4.24758 | 1.853435 |
| H | -2.26973 | -3.85043 | 0.033232 |
| H | -2.09025 | -5.04346 | 1.431774 |
| C | 0.23622 | -2.7286 | 0.294711 |
| H | -0.23153 | -2.53073 | -0.68113 |
| O | 1.611577 | -2.92449 | 0.192776 |
| C | 2.299649 | -1.95358 | -0.602 |
| H | 2.156641 | -0.96188 | -0.15609 |
| H | 1.86126 | -1.93858 | -1.61012 |
| C | -3.69611 | -0.26202 | -0.34169 |
| Cl | -4.88881 | -1.52842 | -0.07376 |
| C | 3.773374 | -2.32013 | -0.65543 |
| H | 4.163346 | -2.35505 | 0.367526 |
| H | 3.872766 | -3.33073 | -1.06649 |
| C | 4.591524 | -1.33056 | -1.49374 |
| H | 4.474024 | -0.32098 | -1.08298 |
| H | 4.188146 | -1.29483 | -2.51296 |
| C | 6.079557 | -1.68791 | -1.5494 |
| H | 6.232525 | -2.67886 | -1.98787 |
| H | 6.638314 | -0.96743 | -2.15261 |
| H | 6.521323 | -1.69733 | -0.54827 |

05. ITX+OCT group TS in Hydrogen Abstraction Reaction

| H | X  0.34107 | Y  1.716124 | Z  1.160445 |
| --- | --- | --- | --- |
| C | -3.27301 | -1.40765 | 2.590455 |
| C | -3.67787 | -2.52535 | 1.845726 |
| C | -2.8638 | -2.99225 | 0.819501 |
| C | -1.65461 | -2.35666 | 0.532287 |
| C | -1.21529 | -1.22393 | 1.280136 |
| C | -2.07444 | -0.77542 | 2.319541 |
| C | 0.030933 | -0.55918 | 1.037202 |
| S | -0.75505 | -2.9632 | -0.85639 |
| C | 0.795679 | -2.13191 | -0.76697 |
| C | 1.023705 | -1.03862 | 0.119356 |
| O | 0.327649 | 0.489406 | 1.822242 |
| C | 2.306507 | -0.43797 | 0.074161 |
| C | 1.792601 | -2.56607 | -1.63901 |
| C | 3.03321 | -1.93888 | -1.65871 |
| H | -3.90372 | -1.03737 | 3.39119 |
| H | -4.61423 | -3.02456 | 2.063812 |
| H | -3.16478 | -3.85576 | 0.236058 |
| H | -1.75154 | 0.077229 | 2.900824 |
| H | 2.491246 | 0.369106 | 0.770876 |
| H | 1.597282 | -3.40129 | -2.30318 |
| H | 3.798846 | -2.28957 | -2.34197 |
| C | 0.869 | 3.685943 | 1.729623 |
| C | 2.142527 | 4.111606 | 1.802527 |
| H | 0.183845 | 3.956015 | 2.531773 |
| H | 2.867744 | 3.864695 | 1.033541 |
| H | 2.489766 | 4.723204 | 2.627101 |
| C | 0.321326 | 2.842654 | 0.663567 |
| H | 0.998239 | 2.751387 | -0.19164 |
| C | -1.62139 | 2.227543 | -0.89601 |
| H | -1.53558 | 1.173835 | -0.61008 |
| H | -0.96341 | 2.364827 | -1.76374 |
| C | 3.30605 | -0.86634 | -0.79007 |
| C | -3.06744 | 2.527656 | -1.30514 |
| H | -3.15716 | 3.587616 | -1.57828 |
| H | -3.72635 | 2.379538 | -0.43978 |
| C | -3.56493 | 1.663935 | -2.4698 |
| H | -3.47318 | 0.605901 | -2.19762 |
| H | -2.90777 | 1.813283 | -3.33529 |
| C | -5.01205 | 1.964659 | -2.87056 |
| H | -5.12875 | 3.008077 | -3.18077 |
| H | -5.33575 | 1.3332 | -3.70271 |
| H | -5.69784 | 1.790066 | -2.03542 |
| C | -1.12482 | 3.109797 | 0.254091 |
| H | -1.77804 | 2.987032 | 1.126608 |
| H | -1.21045 | 4.165926 | -0.04106 |
| C | 4.68216 | -0.21584 | -0.79282 |
| H | 5.264972 | -0.71183 | -1.57734 |
| C | 5.419974 | -0.43828 | 0.540659 |
| H | 6.431243 | -0.02294 | 0.49367 |
| H | 4.895609 | 0.047366 | 1.368506 |
| H | 5.49945 | -1.50274 | 0.775254 |
| C | 4.616157 | 1.280717 | -1.14586 |
| H | 5.622855 | 1.704499 | -1.20975 |
| H | 4.117909 | 1.440196 | -2.10561 |
| H | 4.066966 | 1.841716 | -0.38433 |
|  |  |  |  |

06. ITX+ABE group TS in Hydrogen Abstraction Reaction

| H | X  1.30456 | Y  1.139674 | Z  -1.69413 |
| --- | --- | --- | --- |
| C | 3.878085 | -2.11667 | -0.6718 |
| C | 3.860645 | -2.89237 | 0.496475 |
| C | 2.678791 | -3.00346 | 1.220765 |
| C | 1.526413 | -2.33805 | 0.79953 |
| C | 1.521496 | -1.5283 | -0.37416 |
| C | 2.741676 | -1.4545 | -1.097 |
| C | 0.360128 | -0.82727 | -0.84251 |
| S | 0.099636 | -2.50744 | 1.8221 |
| C | -1.20436 | -1.81216 | 0.863102 |
| C | -0.95446 | -1.02148 | -0.29726 |
| O | 0.464223 | -0.05429 | -1.92855 |
| C | -2.0802 | -0.43293 | -0.92578 |
| C | -3.37589 | -0.60414 | -0.45527 |
| C | -2.50245 | -1.99324 | 1.337274 |
| C | -3.57948 | -1.39889 | 0.687697 |
| H | 4.788881 | -2.0439 | -1.25608 |
| H | 4.751465 | -3.41127 | 0.828993 |
| H | 2.645213 | -3.61261 | 2.117741 |
| H | 2.751294 | -0.87716 | -2.01135 |
| H | -1.88623 | 0.159192 | -1.81042 |
| H | -2.67107 | -2.60388 | 2.217909 |
| H | -4.58343 | -1.55148 | 1.068451 |
| C | -4.56263 | 0.037008 | -1.16049 |
| H | -5.4545 | -0.2201 | -0.5776 |
| C | -4.45867 | 1.572554 | -1.19059 |
| H | -3.59681 | 1.900012 | -1.77893 |
| H | -5.35509 | 2.00839 | -1.64191 |
| H | -4.35207 | 1.981788 | -0.18256 |
| C | -4.75778 | -0.53236 | -2.57815 |
| H | -4.86661 | -1.61964 | -2.55572 |
| H | -5.65414 | -0.10919 | -3.04172 |
| H | -3.9043 | -0.29567 | -3.21976 |
| C | 3.196718 | 2.106838 | -2.12749 |
| C | 3.458444 | 2.38057 | -3.40926 |
| H | 3.984323 | 1.758339 | -1.46509 |
| H | 2.685174 | 2.721232 | -4.09065 |
| H | 4.455687 | 2.267776 | -3.81749 |
| C | 1.853301 | 2.192698 | -1.51955 |
| H | 1.176541 | 2.886603 | -2.0405 |
| O | 1.928645 | 2.446892 | -0.15439 |
| C | 0.672147 | 2.707621 | 0.48176 |
| H | 0.004301 | 1.852864 | 0.322857 |
| H | 0.211779 | 3.58969 | 0.014068 |
| C | 0.915797 | 2.941374 | 1.963238 |
| H | 1.619209 | 3.773228 | 2.079902 |
| H | 1.402531 | 2.054138 | 2.381833 |
| C | -0.37875 | 3.23812 | 2.729607 |
| H | -1.08052 | 2.40698 | 2.594188 |
| H | -0.86411 | 4.122327 | 2.298772 |
| C | -0.14328 | 3.466592 | 4.225431 |
| H | 0.528935 | 4.313309 | 4.395544 |
| H | -1.08079 | 3.675977 | 4.747463 |
| H | 0.308404 | 2.586204 | 4.692607 |

07. TX+CYC group TS in Hydrogen Abstraction Reaction

| H | X  -1.31784 | Y  1.173068 | Z  1.022554 |
| --- | --- | --- | --- |
| C | 1.194473 | -3.244245 | 2.10374 |
| C | 2.256331 | -3.722847 | 1.323561 |
| C | 2.837548 | -2.887949 | 0.374613 |
| C | 2.373157 | -1.583513 | 0.201205 |
| C | 1.303382 | -1.069335 | 0.990974 |
| C | 0.731107 | -1.951414 | 1.945745 |
| C | 0.821291 | 0.275891 | 0.868124 |
| S | 3.089999 | -0.656978 | -1.11714 |
| C | 2.518828 | 0.990395 | -0.84076 |
| C | 1.467589 | 1.281645 | 0.075755 |
| O | -0.1937 | 0.623184 | 1.673931 |
| C | 1.089371 | 2.644849 | 0.194932 |
| C | 3.122538 | 1.995709 | -1.59675 |
| C | 2.70913 | 3.318237 | -1.47154 |
| H | 0.732512 | -3.891827 | 2.840725 |
| H | 2.624119 | -4.733679 | 1.452383 |
| H | 3.658086 | -3.247682 | -0.23672 |
| H | -0.0795 | -1.574932 | 2.554355 |
| H | 0.328316 | 2.899 | 0.919866 |
| H | 3.922099 | 1.738593 | -2.28323 |
| H | 3.182794 | 4.092706 | -2.06252 |
| C | -2.67715 | 2.691292 | 1.589557 |
| C | -2.46771 | 4.006202 | 1.407975 |
| H | -3.08895 | 2.354629 | 2.53965 |
| H | -2.05586 | 4.391283 | 0.480365 |
| H | -2.71339 | 4.730565 | 2.175874 |
| C | -2.36855 | 1.64536 | 0.606457 |
| H | -2.10314 | 2.057596 | -0.37166 |
| C | 1.692946 | 3.634391 | -0.56012 |
| H | 1.385977 | 4.666644 | -0.43192 |
| C | -3.27936 | -0.354206 | -0.75328 |
| C | -2.00252 | -1.191287 | -0.93946 |
| C | -4.39364 | -1.416522 | -0.8627 |
| H | -3.36882 | 0.324967 | -1.61404 |
| C | -2.3649 | -2.172237 | -2.06973 |
| H | -1.79033 | -1.736965 | -0.01277 |
| H | -1.11807 | -0.592503 | -1.16779 |
| C | -3.88926 | -2.443697 | -1.90924 |
| H | -4.5185 | -1.901008 | 0.112763 |
| H | -5.36114 | -0.981432 | -1.12765 |
| H | -2.16626 | -1.707269 | -3.04034 |
| H | -1.76955 | -3.087172 | -2.03096 |
| H | -4.40761 | -2.32187 | -2.86369 |
| H | -4.08322 | -3.466745 | -1.57817 |
| C | -3.36617 | 0.485755 | 0.523298 |
| H | -3.25407 | -0.163104 | 1.40136 |
| H | -4.37856 | 0.908469 | 0.590136 |

08. TX+ACE group TS in Hydrogen Abstraction Reaction

| H | X  -1.350107 | Y  -1.0401 | Z  -0.96944 |
| --- | --- | --- | --- |
| C | 1.417986 | 3.44834 | -1.74185 |
| C | 2.470209 | 3.8158 | -0.89193 |
| C | 2.993696 | 2.87779 | -0.00707 |
| C | 2.479616 | 1.5814 | 0.034021 |
| C | 1.416537 | 1.18109 | -0.82694 |
| C | 0.90589 | 2.16399 | -1.715 |
| C | 0.886646 | -0.1545 | -0.84189 |
| S | 3.114866 | 0.51211 | 1.285909 |
| C | 2.552377 | -1.0888 | 0.795563 |
| C | 1.514412 | -1.2561 | -0.16576 |
| O | -0.140021 | -0.389 | -1.66042 |
| C | 1.131724 | -2.5911 | -0.46135 |
| C | 3.139821 | -2.1842 | 1.428242 |
| C | 2.722163 | -3.4784 | 1.131791 |
| H | 1.001937 | 4.1756 | -2.43027 |
| H | 2.876166 | 4.81987 | -0.91839 |
| H | 3.808291 | 3.14991 | 0.655289 |
| H | 0.104202 | 1.8736 | -2.38052 |
| H | 0.383455 | -2.7446 | -1.22724 |
| H | 3.930111 | -2.0202 | 2.15292 |
| H | 3.183205 | -4.3239 | 1.627693 |
| C | -2.818874 | -2.4944 | -1.50961 |
| C | -2.546876 | -3.8 | -1.56211 |
| H | -3.438946 | -2.0291 | -2.27079 |
| H | -1.930945 | -4.2861 | -0.81212 |
| H | -2.935567 | -4.4253 | -2.35726 |
| C | -2.28505 | -1.574 | -0.47698 |
| H | -1.866832 | -2.0908 | 0.398044 |
| C | 1.719719 | -3.6714 | 0.172026 |
| H | 1.412671 | -4.6778 | -0.09132 |
| C | -2.929713 | 0.2125 | 1.013387 |
| C | -2.247801 | 1.52544 | 0.571453 |
| C | -4.270655 | 0.63417 | 1.6656 |
| H | -2.296185 | -0.3661 | 1.69323 |
| C | -3.398868 | 2.53099 | 0.42496 |
| H | -1.65141 | 1.38707 | -0.32977 |
| H | -1.569722 | 1.85161 | 1.368055 |
| C | -4.316648 | 2.17723 | 1.606146 |
| H | -5.078562 | 0.19768 | 1.07371 |
| H | -4.366921 | 0.24584 | 2.681737 |
| H | -3.059555 | 3.56932 | 0.438673 |
| H | -3.922411 | 2.36649 | -0.5228 |
| H | -3.909594 | 2.60552 | 2.528664 |
| H | -5.334049 | 2.55873 | 1.49394 |
| O | -3.240597 | -0.6268 | -0.11867 |

09. CTX+CYC group TS in Hydrogen Abstraction Reaction

| H | X  0.983097 | Y  -1.51785 | Z  1.189979 |
| --- | --- | --- | --- |
| C | 1.810626 | 3.597633 | 1.744184 |
| C | 1.133258 | 4.558709 | 0.981521 |
| C | 0.026539 | 4.179257 | 0.228308 |
| C | -0.41024 | 2.854442 | 0.233811 |
| C | 0.253332 | 1.861811 | 1.011322 |
| C | 1.381504 | 2.283377 | 1.762649 |
| C | -0.18992 | 0.49837 | 1.073512 |
| S | -1.73409 | 2.453573 | -0.86291 |
| C | -2.23003 | 0.838183 | -0.35143 |
| C | -1.42762 | 0.042017 | 0.516318 |
| O | 0.532149 | -0.33257 | 1.840126 |
| C | -1.92847 | -1.24773 | 0.836113 |
| C | -3.43002 | 0.362697 | -0.87691 |
| C | -3.86063 | -0.91842 | -0.55305 |
| H | 2.677698 | 3.885798 | 2.328016 |
| H | 1.464964 | 5.58996 | 0.972615 |
| H | -0.50352 | 4.914046 | -0.36783 |
| H | 1.893819 | 1.541354 | 2.359506 |
| H | -1.36694 | -1.8548 | 1.532755 |
| H | -4.02611 | 0.986321 | -1.53173 |
| C | 1.2172 | -3.50206 | 1.878718 |
| C | 0.237674 | -4.42143 | 1.912493 |
| H | 1.887187 | -3.42059 | 2.733065 |
| H | -0.45653 | -4.54019 | 1.08641 |
| H | 0.114147 | -5.0872 | 2.758776 |
| C | 1.451703 | -2.56274 | 0.774179 |
| H | 0.843133 | -2.79495 | -0.10514 |
| C | -3.11672 | -1.72367 | 0.31421 |
| H | -3.48211 | -2.70635 | 0.584747 |
| C | 3.155467 | -1.66329 | -0.94734 |
| C | 2.628988 | -0.23433 | -1.16446 |
| C | 4.65029 | -1.5263 | -1.30629 |
| H | 2.682749 | -2.31667 | -1.69545 |
| C | 3.325814 | 0.223286 | -2.45905 |
| H | 2.93617 | 0.395544 | -0.32153 |
| H | 1.54008 | -0.17587 | -1.22808 |
| C | 4.709221 | -0.49014 | -2.4586 |
| H | 5.192164 | -1.1495 | -0.43097 |
| H | 5.104982 | -2.48403 | -1.57359 |
| H | 2.739281 | -0.09479 | -3.32649 |
| H | 3.41439 | 1.310237 | -2.51997 |
| H | 4.894667 | -0.97758 | -3.41901 |
| H | 5.52708 | 0.218097 | -2.30604 |
| Cl | -5.36684 | -1.52054 | -1.22566 |
| C | 2.918474 | -2.27732 | 0.434321 |
| H | 3.359327 | -1.63388 | 1.206359 |
| H | 3.466023 | -3.2287 | 0.488193 |

10. CTX+ACE group TS in Hydrogen Abstraction Reaction

| H | X  1.107885 | Y  1.397179 | Z  -1.07347 |
| --- | --- | --- | --- |
| C | 1.46935 | -3.92907 | -1.44797 |
| C | 0.713311 | -4.77407 | -0.62447 |
| C | -0.35272 | -4.24921 | 0.100287 |
| C | -0.66947 | -2.89401 | 0.00692 |
| C | 0.077939 | -2.01711 | -0.83089 |
| C | 1.158619 | -2.58539 | -1.5536 |
| C | -0.24497 | -0.62442 | -0.98506 |
| S | -1.94425 | -2.30023 | 1.075087 |
| C | -2.33362 | -0.7096 | 0.413778 |
| C | -1.462 | -0.04334 | -0.49569 |
| O | 0.558249 | 0.102993 | -1.76055 |
| C | -1.86903 | 1.244148 | -0.93574 |
| C | -3.51095 | -0.11648 | 0.864512 |
| C | -3.84922 | 1.157646 | 0.422685 |
| H | 2.304241 | -4.33083 | -2.01123 |
| H | 0.952148 | -5.82793 | -0.54771 |
| H | -0.94515 | -4.89356 | 0.740625 |
| H | 1.730735 | -1.93394 | -2.20036 |
| H | -1.253 | 1.749322 | -1.66728 |
| H | -4.16085 | -0.64302 | 1.552548 |
| C | 1.513383 | 3.392337 | -1.71252 |
| C | 0.555783 | 4.300609 | -1.9089 |
| H | 2.352368 | 3.317407 | -2.3987 |
| H | -0.28952 | 4.395378 | -1.23449 |
| H | 0.590128 | 4.983501 | -2.74964 |
| C | 1.506082 | 2.401595 | -0.60786 |
| H | 0.783337 | 2.638311 | 0.185772 |
| C | -3.03527 | 1.836838 | -0.48873 |
| H | -3.33088 | 2.813066 | -0.85215 |
| C | 2.908107 | 1.433191 | 1.102617 |
| C | 3.132839 | -0.06187 | 0.787634 |
| C | 4.18582 | 1.903557 | 1.842252 |
| H | 1.99929 | 1.59271 | 1.691761 |
| C | 4.658618 | -0.2343 | 0.79136 |
| H | 2.647287 | -0.35676 | -0.14218 |
| H | 2.690607 | -0.65779 | 1.593953 |
| C | 5.103298 | 0.665744 | 1.955252 |
| H | 4.653748 | 2.680829 | 1.23365 |
| H | 3.951947 | 2.348071 | 2.811888 |
| H | 4.967562 | -1.27548 | 0.909319 |
| H | 5.079736 | 0.131007 | -0.15127 |
| H | 4.930002 | 0.149582 | 2.905839 |
| H | 6.162733 | 0.929132 | 1.91887 |
| O | 2.791428 | 2.215053 | -0.10468 |
| Cl | -5.32643 | 1.910126 | 1.001722 |

11. ITX+CYC group TS in Hydrogen Abstraction Reaction

| H | X  -1.27073 | Y  -1.48558 | Z  -1.26844 |
| --- | --- | --- | --- |
| C | -2.2971 | 3.595751 | -1.58674 |
| C | -1.60376 | 4.556878 | -0.8377 |
| C | -0.4377 | 4.192696 | -0.17123 |
| C | 0.042667 | 2.885236 | -0.24914 |
| C | -0.63724 | 1.892656 | -1.01489 |
| C | -1.82686 | 2.299 | -1.67707 |
| C | -0.15643 | 0.54938 | -1.14812 |
| S | 1.45041 | 2.498077 | 0.741736 |
| C | 1.960711 | 0.910683 | 0.158207 |
| C | 1.128165 | 0.115305 | -0.6796 |
| O | -0.898 | -0.28187 | -1.89789 |
| C | 1.650163 | -1.14618 | -1.05879 |
| C | 3.204054 | 0.457157 | 0.593132 |
| C | 3.691594 | -0.80091 | 0.227493 |
| H | -3.20999 | 3.870871 | -2.10341 |
| H | -1.96851 | 5.57491 | -0.77281 |
| H | 0.105951 | 4.926612 | 0.413856 |
| H | -2.35269 | 1.55856 | -2.26406 |
| H | 1.067045 | -1.7573 | -1.73425 |
| H | 3.800706 | 1.105576 | 1.226709 |
| C | -1.5242 | -3.44764 | -2.01717 |
| C | -0.52782 | -4.33474 | -2.1774 |
| H | -2.26772 | -3.34959 | -2.8066 |
| H | 0.239516 | -4.46726 | -1.42107 |
| H | -0.46057 | -4.95807 | -3.06153 |
| C | -1.68593 | -2.56226 | -0.85693 |
| H | -1.00083 | -2.81405 | -0.04183 |
| C | 2.886396 | -1.58386 | -0.61626 |
| H | 3.251172 | -2.55296 | -0.94312 |
| C | -3.26011 | -1.7749 | 1.03461 |
| C | -2.75377 | -0.34088 | 1.265366 |
| C | -4.7219 | -1.69392 | 1.522316 |
| H | -2.70997 | -2.44333 | 1.713478 |
| C | -3.35117 | 0.048511 | 2.630423 |
| H | -3.14575 | 0.311119 | 0.476237 |
| H | -1.66538 | -0.25339 | 1.240617 |
| C | -4.70549 | -0.71399 | 2.723536 |
| H | -5.34315 | -1.2907 | 0.713977 |
| H | -5.13205 | -2.67421 | 1.780086 |
| H | -2.68174 | -0.27519 | 3.433379 |
| H | -3.47031 | 1.129068 | 2.736391 |
| H | -4.78264 | -1.25208 | 3.671776 |
| H | -5.55694 | -0.03042 | 2.683519 |
| C | 5.046271 | -1.29911 | 0.703382 |
| H | 5.181176 | -2.29873 | 0.274496 |
| C | 5.103329 | -1.44418 | 2.23572 |
| H | 6.062888 | -1.86826 | 2.546702 |
| H | 4.993119 | -0.47478 | 2.730998 |
| H | 4.307182 | -2.09777 | 2.601149 |
| C | 6.200267 | -0.41822 | 0.188717 |
| H | 7.166323 | -0.83682 | 0.486748 |
| H | 6.183114 | -0.34159 | -0.9013 |
| H | 6.138328 | 0.595569 | 0.595598 |
| C | -3.12471 | -2.32756 | -0.38618 |
| H | -3.64201 | -1.6648 | -1.0917 |
| H | -3.65484 | -3.28921 | -0.43433 |

12. ITX+ACE group TS in Hydrogen Abstraction Reaction

| H | X  1.317027 | Y  1.396948 | Z  -1.16837 |
| --- | --- | --- | --- |
| C | 2.081214 | -3.87114 | -1.32512 |
| C | 1.349778 | -4.74162 | -0.50532 |
| C | 0.21716 | -4.27054 | 0.152955 |
| C | -0.19053 | -2.94568 | -0.00193 |
| C | 0.530856 | -2.04381 | -0.83809 |
| C | 1.682083 | -2.55773 | -1.49213 |
| C | 0.119792 | -0.68532 | -1.05336 |
| S | -1.55157 | -2.40925 | 0.986704 |
| C | -2.02356 | -0.87218 | 0.252843 |
| C | -1.15859 | -0.17605 | -0.63851 |
| O | 0.905924 | 0.072597 | -1.82175 |
| C | -1.64064 | 1.059817 | -1.13677 |
| C | -3.26018 | -0.35103 | 0.62496 |
| C | -3.70886 | 0.881915 | 0.140831 |
| H | 2.967337 | -4.22986 | -1.83709 |
| H | 1.658457 | -5.77253 | -0.3805 |
| H | -0.35716 | -4.93437 | 0.79007 |
| H | 2.2364 | -1.88838 | -2.13613 |
| H | -1.03167 | 1.589335 | -1.85731 |
| H | -3.88317 | -0.92559 | 1.302872 |
| C | 1.651078 | 3.397195 | -1.84229 |
| C | 0.650391 | 4.222552 | -2.15626 |
| H | 2.55043 | 3.3643 | -2.45103 |
| H | -0.25655 | 4.273204 | -1.56205 |
| H | 0.70864 | 4.876944 | -3.01809 |
| C | 1.619815 | 2.445088 | -0.70618 |
| H | 0.822884 | 2.654723 | 0.020899 |
| C | -2.87143 | 1.566 | -0.75577 |
| H | -3.20723 | 2.509583 | -1.17541 |
| C | 2.943674 | 1.613745 | 1.133241 |
| C | 3.308607 | 0.134939 | 0.880342 |
| C | 4.113533 | 2.199325 | 1.963858 |
| H | 1.9784 | 1.713526 | 1.639811 |
| C | 4.83691 | 0.082765 | 1.018833 |
| H | 2.929499 | -0.216 | -0.07888 |
| H | 2.844392 | -0.4776 | 1.661381 |
| C | 5.108621 | 1.039569 | 2.190652 |
| H | 4.572547 | 2.995859 | 1.373564 |
| H | 3.764284 | 2.647135 | 2.896673 |
| H | 5.212979 | -0.92824 | 1.191702 |
| H | 5.309552 | 0.459909 | 0.105701 |
| H | 4.891805 | 0.532099 | 3.137046 |
| H | 6.144268 | 1.383758 | 2.238167 |
| O | 2.87092 | 2.357238 | -0.10155 |
| C | -5.05739 | 1.453599 | 0.546455 |
| H | -5.15936 | 2.414262 | 0.028666 |
| C | -5.13554 | 1.735278 | 2.058698 |
| H | -6.08898 | 2.207864 | 2.313457 |
| H | -5.05788 | 0.811279 | 2.639559 |
| H | -4.32931 | 2.399852 | 2.379606 |
| C | -6.22559 | 0.559159 | 0.09043 |
| H | -7.18509 | 1.025888 | 0.332953 |
| H | -6.19303 | 0.386215 | -0.98814 |
| H | -6.19636 | -0.416 | 0.585783 |
|  |  |  |  |


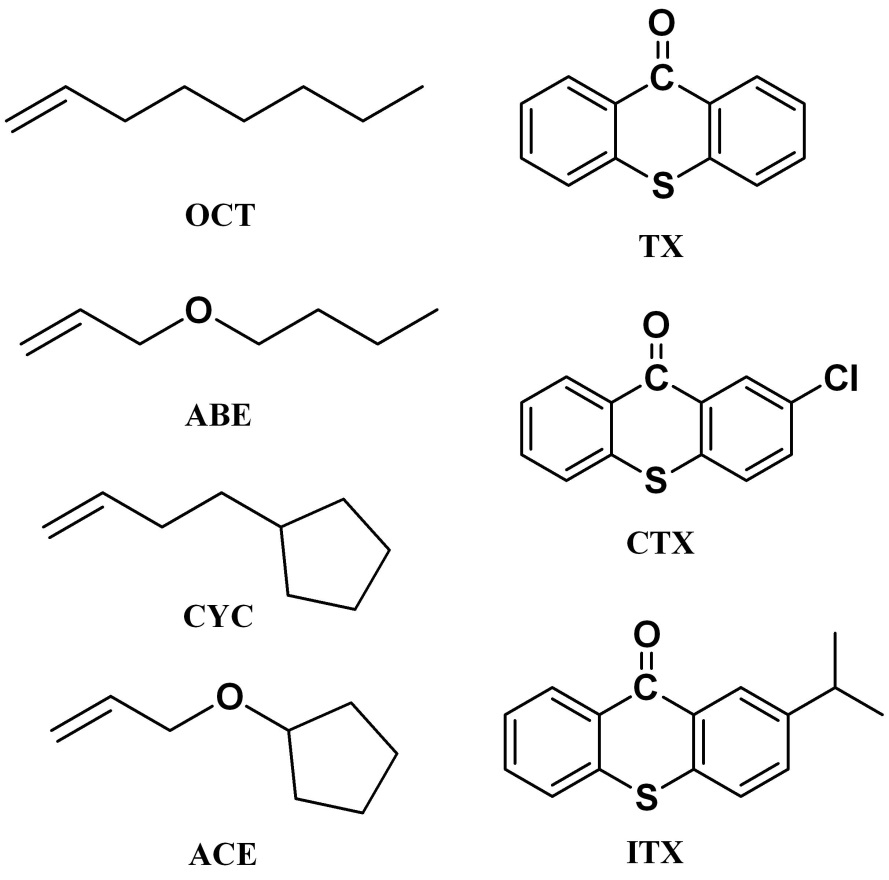


**Figure S1** Chemical structures of the studied donors and acceptors

**Table S1** The values for highest occupied molecular orbital (HOMO) and lowest unoccupied molecular orbital (LUMO) of the OCT, ABE, CYC and ACE at the B3LYP/6-311++g(d,p) level.

|  | **HOMO**  **(eV)** | **LUMO**  **(eV)** | **△*E*_HOMO-LUMO_**  **(eV)** |
| --- | --- | --- | --- |
| OCT | -8.53 | -0.02 | 8.51 |
| ABE | -8.76 | -0.03 | 8.73 |
| CYC | -8.51 | -0.05 | 8.46 |
| ACE | -8.65 | -0.07 | 8.58 |

**Table S2** Changes in bond angles and distances between the reaction complex (RC) and the transition state (TS).

|  |  | α _TS_ | α _Prod_ | β _TS_ | β _Rct_ | d _TS_ | d _Prod_ |
| --- | --- | --- | --- | --- | --- | --- | --- |
| TX+ | OCT | 119.10 | 120.04 | 32.85 | 33.34 | 1.42 | 0.97 |
|  | ABE | 119.11 | 120.44 | 30.70 | 38.45 | 1.52 | 0.97 |
| CTX+ | OCT | 119.24 | 120.11 | 34.85 | 35.34 | 1.43 | 0.97 |
|  | ABE | 119.27 | 120.45 | 32.65 | 40.49 | 1.53 | 0.96 |
| ITX+ | OCT | 118.82 | 119.69 | 38.75 | 35.88 | 1.39 | 0.97 |
|  | ABE | 118.98 | 120.09 | 22.39 | 14.93 | 1.48 | 0.97 |
| TX+ | CYC | 119.42 | 120.22 | 39.80 | 41.62 | 1.41 | 0.97 |
|  | ACE | 119.36 | 120.46 | 45.86 | 54.89 | 1.54 | 0.96 |
| CTX+ | CYC | 119.35 | 120.18 | 39.36 | 41.63 | 1.43 | 0.97 |
|  | ACE | 119.26 | 120.42 | 45.21 | 53.72 | 1.56 | 0.97 |
| ITX+ | CYC | 119.31 | 120.17 | 40.16 | 41.85 | 1.41 | 0.97 |
|  | ACE | 119.25 | 120.52 | 46.03 | 54.67 | 1.53 | 0.96 |

**Table S3** The rate constants value with Eckart’s tunneling correction as a function of temperature from 500 K to 2500 K.

|  |  | 500k | 1000k | 1500k | 2000k 2500k | | |
| --- | --- | --- | --- | --- | --- | --- | --- |
| TX | OCT | 8.02E-17 | 4.48E-15 | 3.25E-14 | 1.17E-13 | 3.02E-13 |  |
|  | ABE | 1.46E-15 | 1.85E-14 | 7.89E-14 | 2.09E-13 | 4.35E-13 |  |
|  | CYC | 1.03E-18 | 9.12E-16 | 7.84E-15 | 3.09E-14 | 8.27E-14 |  |
|  | ACE | 4.23E-16 | 6.78E-15 | 2.96E-14 | 8.60E-14 | 1.83E-13 |  |
| CTX | OCT | 6.50E-17 | 3.73E-15 | 2.74E-14 | 9.91E-14 | 2.56E-13 |  |
|  | ABE | 1.02E-15 | 1.52E-14 | 6.75E-14 | 1.85E-13 | 4.00E-13 |  |
|  | CYC | 9.40E-18 | 5.94E-16 | 4.50E-15 | 1.66E-14 | 4.31E-14 |  |
|  | ACE | 5.31E-16 | 6.89 E-15 | 2.69 E-14 | 7.31E-14 | 1.58 E-13 |  |
| ITX | OCT | 7.41E-17 | 7.10E-15 | 6.35E-14 | 2.56E-13 | 6.97E-13 |  |
|  | ABE | 1.32E-15 | 3.01E-14 | 1.57E-13 | 4.72E-13 | 1.10E-12 |  |
|  | CYC | 7.49E-18 | 6.77E-16 | 5.86E-15 | 2.32E-14 | 6.23E-14 |  |
|  | ACE | 2.64E-16 | 4.29E-15 | 1.90E-14 | 5.57E-14 | 1.18E-13 |  |
